# Reference Ranges of 8-Isoprostane Concentrations in Exhaled Breath Condensate (EBC): A Systematic Review and Meta-Analysis

**DOI:** 10.3390/ijms21113822

**Published:** 2020-05-28

**Authors:** Yara Shoman, Pascal Wild, Maud Hemmendinger, Melanie Graille, Jean-Jacques Sauvain, Nancy B. Hopf, Irina Guseva Canu

**Affiliations:** 1 Center for Primary Care and Public Health (Unisanté), University of Lausanne, 1066 Epalinges-Lausanne, Switzerland; Pascal.Wild@inrs.fr (P.W.); maud.hemmendinger@unisante.ch (M.H.); Melanie.Graille@unisante.ch (M.G.); jean-jacques.sauvain@unisante.ch (J.-J.S.); nancy.hopf@unisante.ch (N.B.H.); irina.guseva-canu@unisante.ch (I.G.C.); 2 Institut national de recherche et de sécurité (INRS), 54500 Vandœuvre-lès-Nancy, France

**Keywords:** 8-isoprostane, lung, inflammation, oxidative stress, exhaled air, reference range, healthy adult, exposure, baseline value, gender

## Abstract

Isoprostanes are physiopathologic mediators of oxidative stress, resulting in lipid peroxidation. 8-isoprostane seems particularly useful for measuring oxidative stress damage. However, no reference range values are available for 8-isoprosante in exhaled breath condensate (EBC) of healthy adults, enabling its meaningful interpretation as a biomarker. We conducted this systematic review and meta-analysis according to the protocol following PROSPERO (CRD42020146623). After searching and analyzing the literature, we included 86 studies. After their qualitative synthesis and risk of bias assessment, 52 studies were included in meta-analysis. The latter focused on studies using immunological analytical methods and investigated how the concentrations of 8-isoprostane differ based on gender. We found that gender had no significant effect in 8-isoprostane concentration. Among other studied factors, such as individual characteristics and factors related to EBC collection, only the device used for EBC collection significantly affected measured 8-isoprostane concentrations. However, adjustment for the factors related to EBC collection, yielded uncertainty whether this effect is due to the device itself or to the other factors. Given this uncertainty, we estimated the reference range values of 8-isoprostane stratified by gender and EBC collection device. A better standardization of EBC collection seems necessary; as well more studies using chemical analytical methods to extend this investigation.

## 1. Introduction

Oxidative stress (OS) is an imbalance between the production of reactive oxygen species (free radicals) and antioxidant defenses, which may lead to cell injury [1]. The reactive oxygen species (ROS) can target various substrates in the cell, causing protein, DNA, RNA oxidation, or lipid peroxidation [2]. 8-isoprostane is a lipid peroxidation product of arachidonic acid and was identified through coordinated experimental studies led by the U.S National Institute of Environmental Health Sciences as the most useful biomarker of oxidative damage [3]. It is detectable in exhaled breath condensate (EBC) of healthy subjects and reflects oxidative stress in the respiratory tract [4]. EBC collection is a noninvasive sample collection and can be repeated frequently within short intervals and without adverse events. EBC content analysis has a good reproducibility for several biomarkers of oxidative stress, including pH, H2O2, adenosine, and 8-isoprostane [5]. Several analytical methods for 8-isoprostane for biological matrices have been developed [6], such as mass spectrometry (chemical methods) and immunological methods [7,8]. 8-isoprostane has vasoconstrictive and inflammatory properties and therefore it may serve as a pathologic mediator of oxidative stress [9].

8-isoprostane may be particularly useful for measuring oxidative stress damage. However, no reference ranges are available for 8-isoprosante in EBC of healthy adults, enabling its meaningful interpretation as a biomarker. A narrative review by Koutsokera et al. [10] summarized some values, but to establish the reference range, a stringent methodology comprising systematic review and meta-analysis [11] is required. Thus, we conducted a systematic review and meta-analysis to assess 8-isoprotane concentrations in EBC in a healthy adult population (i.e., aged > 18 years, without diseases, and not exposed to any specific exposure (e.g., occupational exposures such as welders). Gender has influence on oxidative stress responses [12,13]. It was demonstrated that the oxidative stress biomarkers are higher in healthy young males compared to age-matched females [14]. Moreover, Kurti et al. reported that gender differences exist in airway 8-isoprostane responses following an exhaustive exercise in older adults [15]. Therefore, we aimed at investigating the relation between 8-isoprostane concentrations in EBC and gender, but also age, body mass index (BMI), smoking status, the device used during EBC collection, the duration of EBC collection, the storage temperature, and whether the participants were wearing a nose-clip or not. 

## 2. Results

### 2.1. Study Selection

**Figure 1:**
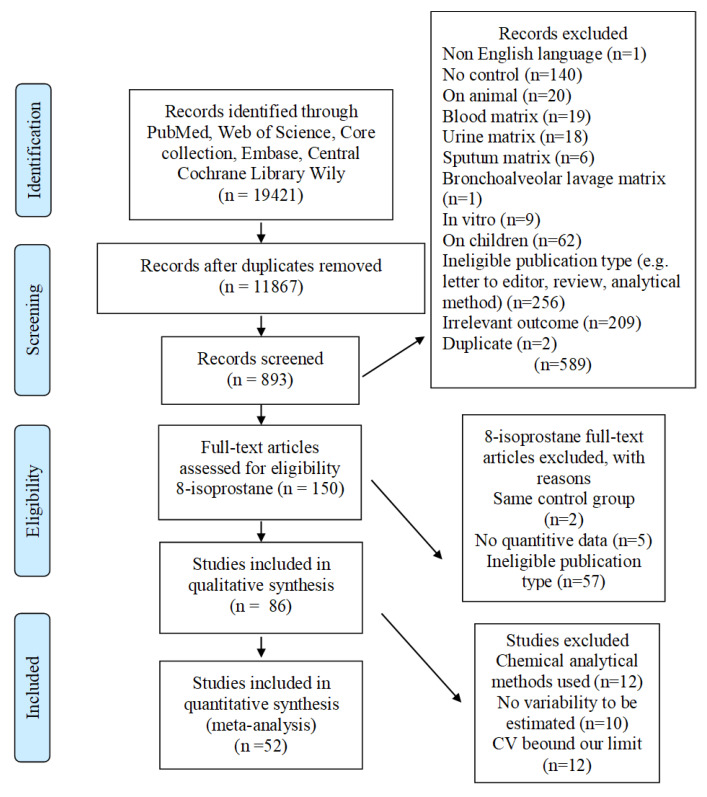
Flow chart of study selection.

We identified 19,421 studies applying the literature search string in four databases (Figure 1), of which 11,867 studies remained after removing duplicates. Restricting the search to include only EBC, gave 893 records. We excluded 548 studies based on the title and abstract screening. Finally, we retained 86 studies where 8-isoprostane was analyzed in EBC. After reading these studies thoroughly, all 86 studies were included in the qualitative synthesis including risk of bias and quality assessment [4,15,16,17,18,19,20,21,22,23,24,25,26,27,28,29,30,31,32,33,34,35,36,37,38,39,40,41,42,43,44,45,46,47,48,49,50,51,52,53,54,55,56,57,58,59,60,61,62,63,64,65,66,67,68,69,70,71,72,73,74,75,76,77,78,79,80,81,82,83,84,85,86,87,88,89,90,91,92,93,94,95,96,97,98,99], and 52 studies into the quantitative analysis [4,15,17,18,19,20,21,22,23,25,26,28,29,30,31,35,38,39,40,44,46,47,48,49,50,51,52,53,54,55,57,59,60,62,63,64,74,75,77,79,80,81,82,84,85,86,88,89,90,93,95,99]. In the quantitative analysis, ten studies were excluded because no information was provided to estimate variability (GSD). Another 12 studies were excluded because their coefficients of variation (CV) were beyond acceptance limits (greater than 10% and lower than 200%). Only 12 studies out of the remaining 64 reported using chemical analytical methods. We therefore decided to include only studies using immunological methods. This resulted in 52 studies for the meta-analysis.

### 2.2. Descriptive Analysis

Table 1 and supplementary Table S1 summarize the 86 studies included in the systematic review. These studies were conducted between 1999 and 2019 in different countries, namely USA, UK, Japan, China, Poland, Czech Republic, Finland, France, Belgium, Sweden, Taiwan, Italy, Turkey, Egypt, Israel, Iceland, and Chile. The included studies had more male participants (59%) than females (41%) and the participants had mean age 46.84 years with standard deviation 2.10. Table 1 and supplementary Table S1 provide detailed information on the quality level of each study. In overall, 15 studies (17.4%) were classified of low quality (high risk of bias), 57 (66.3%) of moderate quality (moderate risk of bias), and only 13 studies (15.1%) were of high quality (low risk of bias). Most of studies lacked the representative sample, they included small number of participants, and used convenience sampling. The other drawbacks were biases in the measurement of the exposure or outcome that resulted in a high risk of bias.

### 2.3. Meta-Analysis

**Figure 2:**
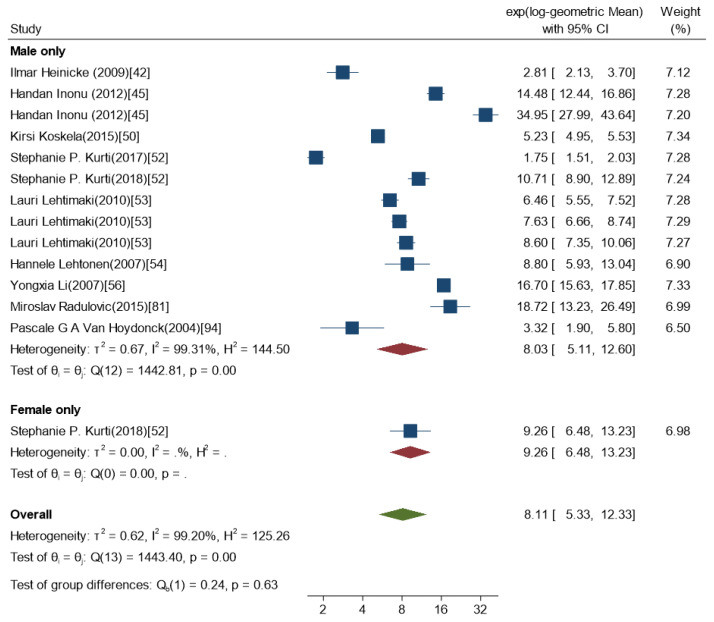
Forest-plot of the 8-isoprostane levels [pg/mL] measured in the exhaled breath condensate using immunological analytical methods and in studied with only females or only males (*n* = 13).

**Figure 3:**
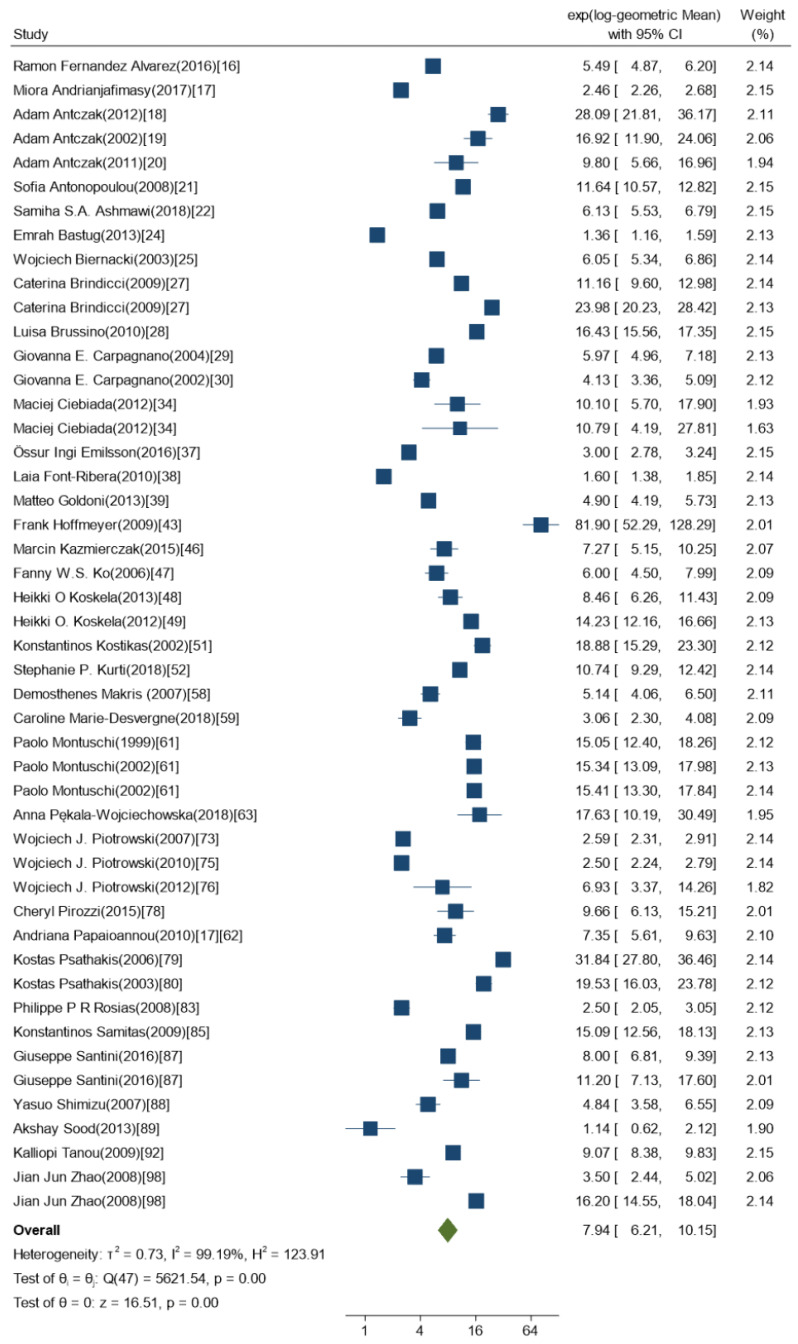
Forest-plot of the 8-isoprostane levels [pg/mL] measured in the exhaled breath condensate using immunological analytical methods and in studies with both males and females (*n* = 39).

We conducted the meta-analysis on healthy subjects aged 18+ years, exclusively. Participants were divided into subgroups depending on their gender. In total, within the 52 studies included in the meta-analysis, we considered 62 subgroups of participants included, with 1980 participants in overall. We had 322 participants in the males only subgroup, and 12 participants in the females only subgroup. The mean age of the subgroups was 44.90 years with standard deviation (SD) 11.61. Participants were 54% males and 46% females. Only 18 studies reported BMI [15,17,18,22,25,31,38,43,47,48,53,57,64,82,84,86,90,95]. Among them 65% reported BMI higher than 25. The between-study heterogeneity was very high (I^2^ = 99.22%) and the mean 8-isoprostane concentration was 7.97 pg/mL with a 95%-confidence interval (95%CI) between 6.46 and 9.85. The heterogeneity remained high even after stratifying by gender, I^2^ = 99.19% for studies with mixed gender groups (both males and females) (Figure 2) and I^2^ = 99.20% for studies with specific gender groups (only males or females) (Figure 3). Due to the high heterogeneity, a meta-regression of all the study groups mostly reflected differences between studies rather than any actual effect of determinants considered. For that reason, a mixed model with study ID as a random effect appeared the most relevant.

Our results showed no significant difference in the 8-isoprostane concentrations in EBC of healthy subjects with respect to gender, age, BMI, or smoking status (Table A1). However, there was a significant difference related to the device used in EBC collection. Measured concentrations were higher when EcoScreen device was used for EBC collection: GM (95%CI): 7.67 pg/mL (5.58–9.76) compared to Rtube device 3.42 pg/mL (0.57–6.27). In nine studies, the name of the device used was not reported [19,20,23,43,52,53,80,81], one study used TurboDECCS [40], and two studies used home-made device [4,62]. In these studies, the concentrations of 8-isoprostane was 14.01 pg/mL (7.03–20.99). It is worth to mention, that after adjusting this model for other factors characterizing the EBC collection and storage, the effect of device became statistically non-significant. None of adjustment factors had significant effect per se (Table A2), which raises question of their importance in frame of standardization guidelines. Table 2 shows 8-isoprostane concentrations when measured using different devices and stratified by gender but the results showed no significant difference.

## 3. Discussion

### 3.1. Interpretation of Results

Several reviews aimed at setting a reference value for 8-isoprostane in EBC in sick populations [3,100]. This is the first study that attempts to determine 8-isoprostane concentration ranges among healthy adults. Moreover, we considered gender and many other factors susceptible to affect the concentration of 8-isoprostane in EBC, including age, smoking status, BMI, collection time of EBC sample, whether the participants were wearing a nose-clip or not, storage of the EBC sample. The only factor that influenced the levels of 8-isoprostane was the type of device used during EBC collection but after adjusting for other determinants during EBC collection, it was not clear if the significant difference was due to the difference in the device itself or the other conditions during EBC collection.

#### 3.1.1. Gender and Individual Characteristics

Gender differences have been observed in oxidative stress responses [13]. We therefore analyzed 8-isoprostane levels according to gender but found no statistically significant difference between males and females. There were only nine studies out of 52 [15,24,46,53,54,55,57,82,95] which focused on the gender issue. This limited number of studies that included separately male and female participants may contribute to the absence of a gender effect on 8-isoprostane concentration in EBC. Furthermore, no difference in 8-isoprostane EBC concentrations were found according to age, smoking status or BMI. A plausible explanation to these findings can be the high level of heterogeneity between studies, which overcame within-study individual variability. We found contradictory results for the correlation between BMI and airway 8-isorprostane, Komakula et al. suggested that BMI alone is not sufficient to produce airway changes in 8-isoprosane airway levels. On the contrary, another study by Samitas et al. found a significant correlation between 8-isoprostane and BMI in healthy controls. Two studies analyzed the influence of age on levels of EBC biomarkers [101,102]. Despite conflicting results, Cruz et al. recommended to take age into account when measuring 8-isoprostane concentrations in healthy population [36]. We followed his recommendation, but observed no difference before and after adjustment for age. Smoking was also shown to affect the level of 8-isoprostane in EBC [46,103]. However, our results failed to indicate any relation between smoking and 8-isoprostane EBC concentrations. The same concerns the BMI. One possible explanation of our finding is that most original studies included in our meta-analysis were of moderate and low quality, due to methodological limitations, and their low quality of evidence. Most of the studies included unrepresentative samples and used convenience sampling method, another main drawback is that some studies did not control the confounding factors between subgroups.

#### 3.1.2. Factors Related to EBC Sample Collection, Storage and Analysis

We presented the healthy population values of 8-isoprostane concentrations based on the type of device used for EBC collection because it was the only factor that significantly changed the values of 8-isoprostnante in EBC. Ahmadzai et al. compared the values of 8-isoprostane in EBC measured by Rtube and EcoScreen, and found no significant difference between the two devices [104]. After adjusting for other elements of the EBC collection process, such as the participants wearing a nose clip or not, duration of the sample collecting process, time of the sample collection and the storage temperature of the sample, there was uncertainty whether the significance difference is derived from the device itself or those factors. The difference between Ecoscreen and Rtube may be due to the different materials or the temperature of the collecting system [105]. Ecoscreen has a Teflon coating, which is considered as a disadvantage [85] despite its high recovery in terms of EBC volume per minute. The duration of EBC collection using Ecoscreen is 10–15 min and it maintains a temperature of −10 °C. RTube is made from polypropylene and needs shorter collection time compared to Ecoscreen. However, RTube sensitivity to the higher ambient temperature constitutes disadvantage [106]. Currently there is no evidence whether wearing a nose-clip or maintaining a particular storing temperature of the EBC sample affect the levels of 8-isoprostane. Regarding the EBC collection duration, Carpenter et al. concluded that the 8-isoprostane concentrations did not differ with longer collection durations [7]. Regarding the assays currently available to measure 8-isoprosante concentrations in EBC, there are chemical method with gas or liquid chromatography coupled with mass spectrometry and immunoassay method. Immunoassays are more accessible for laboratories because they do not require a special apparatus like mass spectrometry [107], but the latter is more sensitive and specific compared to immunoassays [5]. This may explain why most studies used the immunological assays. Usage of chemical method should be encouraged to allow our meta-analyses to be extended to studies using both methods, providing reference ranges for each of them.

### 3.2. Contribution of the Results to the Currently Available Guidelines for EBC Handling

Evidence regarding factors related to EBC collection, storage and analysis is currently very limited, maybe because the EBC is still a relatively new technique. The American Thoracic Society (ATS) published general guidelines for EBC measurement in 2005 [5], and most studies included in our meta-analysis were in compliance with these recommendations. The recommendations were that participants are advised to wear a nose-clip and breathe tidally for generally 10 min and the collected samples should be stored immediately at −70 °C. It was also recommended to report with precise description what type of condensing device is used. Nevertheless, the ATS concluded that every biomarker should be evaluated by the investigators involved taking into account different factors such as the biomarker sensitivity to temperature, the study set-up that requires more than 10 min collection duration, and they should report the route of inhalation (oral or nasal inhalation), and the use of nose-clips [108]. The studies included in our review respected these guidelines regarding the detailed reporting of EBC device features. However, the studies were less compliant with respect to other recommendation e.g., the storage temperature, nose-clips and collection duration, which can explain the high heterogeneity observed between studies. We did not observe any significant difference related to these factors in our review, and our results indicate that further research is needed to develop these recommendations for EBC handling. The guidelines were updated in 2017 [109], the main changes were revising the tidal breathing for a specific duration because it resulted in highly variable volume. The volume exhaled per time (i.e., minute volume) is considered the most significant factor in EBC volume and that is why it should be reported. They also changed the storage temperature from −70 °C to −80 °C. Finally, they concluded that one standardization will not be sufficient to fulfill the requirements of the various biomarkers measured in EBC, and future studies should rely on the inclusion of a systematic and diligent description of the methods and techniques used to collect, store, and analyze EBC.

### 3.3. Strengths and Limitations

This is the first systematic review and meta-analysis on 8-isoprostane in the EBC aiming to estimate references range values for this molecule. Such values are paramount for introduction of 8-isoprostane into clinical and field research and practice, and its further validation as biomarker of oxidative stress damage at pulmonary level. This study was conducted according to the high research standards, as detailed in the standardized research protocol [110]. It is based on an extensive research in four major databases and we believe it is exhaustive. For every record, the screening, review, and data extraction were double-checked by different reviewers independently, and very few discrepancies were observed, all solved by consensus. Nevertheless, this study has also some limitations which deserve discussion. We failed to confirm some individual (gender, age, BMI, and smoking status) or external technical factors (sample collection duration, the use of nose-clips, and the sample storage temperature) hypothesized as determinants of 8-isoprostane concentration in EBC. The most plausible explanation for it is that original studies included into analysis were of low methodological standards. The high between-study heterogeneity observed in the meta-analysis, regardless of the study grouping strategy, is another limitation, mainly due to the low degree of methodological standardization in most studies. Though most studies reported their compliance with guidelines for EBC collection [5], it was often difficult or impossible for us to assess this compliance. Many studies did not report sufficient details on the experimental and analytical procedure, enabling this assessment.

## 4. Materials and Methods

This study was conducted according to the protocol registered with the International Prospective Register of Systematic reviews (registration number CRD 42019124621) [110], and described in detail by Hemmmindinger et al. [111]. Our study is reported following recommendations from Preferred Reporting Items for Systematic Reviews and Meta-Analyses (PRISMA) [112,113].

### 4.1. Literature Search

We conducted literature searches for publications since journal inception and up to 26 March 2019 in the following bibliographic electronic databases: The Cochrane Central Register of controlled Trials (CENTRAL, Cochrane Library), EMBASE, PubMed, and Web of Science. Further details and the complete search strategy (exemplified by the search in PubMED and EMBASE databases) are available as a supplementary digital content. The full search strategy including the search string used can be found in (https://www.doi.org/10.16909/dataset/17) [110]. We searched for different 8-isoprostane synonyms such as F2-isoprostane, 8-Epi-prostaglandin-F2alpha, and 15-f2t-isop.

To be included, articles had to be original research studies, involving healthy adult participants (aged 18+), collecting EBC samples for 8-isoprostane quantification, and published in English or French language. Our exclusion criteria were as follows: studies without quantitative data for 8-isoprostane, non-human studies, reviews, correspondence, conference papers, expert opinions, and editorials, as well as abstracts without full text. Furthermore, we also excluded studies where EBC collection device failed to meet American Thoracic Society and European Respiratory Society methodological recommendations [5]. The recommendations were that participants wear a nose-clip and breathe for generally 10 min and the collected samples should be stored at −70 °C.

Two reviewers (MH and YS) independently performed a first screening of titles and abstracts retrieved during the searches, using Rayyan software [114]. Abstracts with insufficient information with regard to the inclusion and exclusion criteria were downloaded in the EndNote software for a full-text screening. The same reviewers independently assessed each article. Disagreements on the inclusion/exclusion of studies between two reviewers were discussed and solved by consensus; when necessary a third reviewer (IGC) was consulted to reach consensus.

### 4.2. Data Extraction

We used the standardized data extraction form developed as part of the study protocol [111]. When data on several subgroups within a study were available, all subgroup-specific data were extracted. Afterwards, we excluded groups with diseases and groups that were exposed to specific occupational exposures. We kept only the baseline measurement in case the study reported different measurements. We also excluded the studies with identical control groups but we kept the latest and the most complete study. A statistician (PW) crossed-checked all the data extracted for meta-analysis.

### 4.3. Quality Assessment

We used the standardized quality assessment checklist developed as part of the protocol and used in our previous studies [111,115]. This checklist includes four domains: (i) quality of the study sample, (ii) quality of study design and risk of bias, (iii) technical and analytical quality (i.e., quality of biological sample collection and conservation and of the laboratory analyses, and (iv) quality of the data processing, analysis and results’ reporting. Each domain can be assessed separately, based on a number of objective criteria (Supplementary Table S1), and graded by assigning a discreet sub-score value. The resulting sub-scores values can be further summarized in a final score for each study, as recommended in the GRADE guidelines [116]. The total quality scores ranged between 9 and 27. Quality scores lower or equal to 13 corresponded to a low quality of evidence; scores between 14 and 19 to a moderate quality of evidence, and scores higher than 20 to a high quality of evidence. The quality assessments of the included studies were performed by one reviewer (YS). A second reviewer (IGC) independently assessed the quality of 10% of studies, selected randomly as part of the quality control procedure. No discrepancies were found.

### 4.4. Statistical Analysis

We aimed to determine the reference range values of 8-isoprosante in EBC of healthy adults. In the meta-analysis, we calculated geometric means (GM) and geometric standard deviations (GSD) because the values of 8-isoprosante were generally log-normally distributed [115]. The heterogeneity of the original data corresponded to GM and GSD. We followed the instructions of the standard practice in meta-analysis [117], and we represented the data as forest plots including the I-square statistics that estimates the percentage of the between-study heterogeneity. When I-square is high, we used mixed model with study ID as a random effect. Our results were provided on the study subgroup level, rather than on the individual level. We used STATA, version 16 software for data management and statistical analyses.

## 5. Conclusions

This study sets reference range values of 8-isoprostne concentration in EBC of healthy adults depending on different devices used during EBC collection and based on studies using immunological analytical methods. Further research is needed to address gender differences, the effect of age, BMI, smoking status on 8-isoprostne concentration in EBC. Further research should also investigate whether the choice of device affects the measured concentration or whether other determinants, such as the collection duration, the sample storage temperature, and whether the participants are wearing a nose-clip or not determine it and to which extent. The latter is crucial for updating the current recommendations on standardization of EBC collection and measurement of the biomarkers, fundamental for drawing conclusions.

## Figures and Tables

**Table 1:** Description of the included high quality studies.

Author, Year, Country, [Ref]*	Study Objectives	Population Studied and Number of Participants	Control Population and Sub-Population	Method of EBC Collection and Analysis	Main Findings	Quality Score
Antonopoulou,2008, Greece, Ref [21]*	To assess airway inflammation by measuring the levels of 8-isoprostane, interleukin-6 (IL-6), Tumor Necrosis Factor-alpha (TNF-a), and pH in EBC and study their plausible relation with plasma levels of leptin.	112 consecutive patients referred with symptoms suggestive of OSA. After a full night diagnostic polysomnography, 45 OSA patients (37 males, age 52 ± 12 years, BMI 33.5 ± 7, 28 smokers) finally formed the patients group. Patients with Apnea/Hypopnea Index (AHI) 10 were included in the study.	25 healthy subjects non-randomly selected, matched for age, gender, and BMI, (18 males, age 51 ± 7 years, BMI 31 ± 3, 15 smokers). They were mainly recruited from a population used as healthy subjects in other studies of this research group.§	EBC was collected by using a condenser (EcoScreen; Jaeger, Wurzburg, Germany). The condensate was stored at −70 °C.	Increased levels of leptin were not associated with the observed airway inflammation in OSA. The observed airway inflammation seemed to be independent of smoking habit with limited association with disease severity.	20
Bastug, 2013, Turkey, Ref [24]*	To measure oxidative stress in Hyperthyrod patients in EBC through measuring the levels of 8-isoprostane.	42 Hyperthyrodism patients (12 males, 30 females).	42 (12 males, 30 females, non-smokers) age and BMI matched healthy control subjects.§	EBC was collected using a condenser (EcoScreen). Subjects were asked to breath tidally for 15 min using a noseclip. Condensates were stored immediately at −70 °C.	8-isoprostane levels in EBC of hyperthyroid patients were found to be significantly higher than that in healthy control group.	20
Chow, 2009, Australia, Ref [32]	To assess lung oxidative stress and inflammation in vivo in subjects with asbestos-related disorders and compare them with age matched controls.	All subjects (*n* = 60) had a confirmed history of workplace asbestos exposure other than controls (*n* = 26) and were classified into three groups (asbestosis, diffuse pleural thickening (DPT) and pleural plaques (PPs). Smokers were excluded.	Age and sex-matched controls (*n* = 26). All control subjects were never or exsmokers without any evidence of asbestos-related or other lung disease after screening.	EBC was collected using Ecoscreen, subjects breathed tidally with nose-clip on. Condensate was collected after 10 min, the cooled condensate was immediately stored at −80 °C.	In asbestos-related disorders, markers of inflammation and oxidative stress are significantly elevated in subjects with asbestosis compared with healthy individuals but not in pleural diseases.	20
Chow, 2012, Australia, Ref [33]	To investigate whether levels of Several reactive oxygen species (ROS) and Several reactive nitrogen species (RNS) in EBC of patients with PF differed significantly from age- and sex-matched controls, and whether these correlated with lung function.	20 subjects had pulmonary fibrosis (PF).	20 were normal controls (16 male, mean age ± SD 55.3 ± 13.4).	EBC was collected using Ecoscreen, subjects breathed tidally with nose-clip on. Condensate was collected after 10 min, the cooled condensate was immediately stored at 80 °C.	Inflammatory and oxidative stress biomarkers are raised in patients with PF compared with controls. EBC may be useful for detecting and monitoring lung inflammation in PF.	21
Emilsson, 2016, Iceland, Ref [37]*	To investigate the association between nocturnal gastroesophageal reflux (nGER) and respiratory symptoms, exacerbations of respiratory symptoms, lung function and Sleep-disordered breathing (SDB).	This study is based on a 20 years prospective, population-based cohort study in Iceland. Among the 522 subjects contacted, a total of 455 participated, or 87% of those invited. Of the 455, 82 had symptoms suggestive of nGER. These 82 subjects were invited for a second visit in 2013, of which 71 (87%) participated.	Age and gender paired controls without any nGER symptoms (participation rate 78%, *n* = 42, Female 48%, mean age ± SD 56.4 ± 7.0).§	EBC samples were collected with ECoScreen II. Participants wore a nose-clip and used tidal breathing for 15 min. The samples were immediately frozen at −20 °C, and within four hours moved to −80 °C for storage.	In a general population sample, nGER is associated with symptoms of asthma and bronchitis, as well as exacerbations of respiratory symptoms. In addition, nGER is associated with increased respiratory effort during sleep.	21
Hoffmeyer, 2012, Germany, Ref [44]	To evaluate subclinical changes in otherwise healthy current welders with the majority practicing this profession for decades.	58 welders (all male, 27 smokers) from the cross-sectional study WELDOX were examined. Welders were processing mild steel applying gas metal arc welding with solid wire (GMAW) or flux cored wire (FCAW).	NA	EBC was collected after shift with the commercially available temperature-controlled device ECoScreen2. The collection time was exactly 10 min.	An enhanced irritative effect in the lower airways of mild steel welders due to the application of FCAW compared to GMAW, most likely associated with a higher emission of welding fumes.	21
Inonu, 2012,Turkey, Ref [45]*	To evaluate the differences in the burden of oxidative stress in patients with COPD, smokers, and non-smokers by measuring H2O2, MDA, and 8-isoprostane levels in the EBC samples.	The subjects in Group I (*n* = 25) had COPD (all ex-smokers).	Group II (*n* = 26) were healthy smokers (mean age ± SD 61.2 ± 6 y, all males) and Group III (*n* = 29) were healthy nonsmokers (mean age ± SD 60 ± 8 y, all males).§	EBCs were collected using a condenser (EcoScreen). The subjects were asked to breathe while wearing a nose clip, for a period of 15 min. The samples were immediately stored at 70 °C. All EBC samples were collected between 2 PM to 4 PM.	Even if respiratory function tests are within normal limits, oxidant burden in lungs of smokers is equivalent to that in COPD patients. 8-isoprostane could be useful in assessing symptom severity and health status of COPD patients.	23
Lehtimaki, 2010, Finland, Ref [53]*	To find out if borderline parenchymal changes on HRCT in subjects with moderate to heavy asbestos exposure are related to the degree of pulmonary inflammation.	Of the 104 asbestos-exposed men recruited,33 were excluded based on the exclusion criteria. 35 subjects had normal parenchymal findings on HRCT and 31 subjects had borderline parenchymal changes.	41 healthy men (mean age 63) not exposed to asbestos or other harmful agents.§	EBC was collected during 15 min of tidal breathing with Ecoscreen condenser while wearing noseclips. The samples were stored at −70 °C.	Borderline parenchymal changes on HRCT in asbestos-exposed subjects are associated with increased markers of pulmonary inflammation. Such borderline parenchymal changes are likely a mild or early form of the same pathological process that leads to asbestosis.	20
Pelclova, 2007, Czech Republic, Ref [65]	To measure 8-isoprostane, leukotrienes B4, C4, D4, and E4 in exhaled breath condensate in patients with silicosis.	Patients with silicosis (*n* = 60, 58 men and 2 women).	The control group was composed of 25 subjects (23 men and 2 women), previously working as office employees and safety inspectors, never occupationally exposed to fibrogenic dusts.	EBC samples were collected using the EcoScreen. Each subject was asked to breathe through the collection kit for 15 min with more than 2 mL of EBC collected. Samples were immediately frozen after collection (−80 °C)	No significant effect of smoking or alcohol consumption on the markers examined was seen. This is the first study using exhaled breath condensate analysis in patients with silicosis.	20
Pelclova, 2008, Czech Republic, Ref [64]	To investigate the hypothesis that oxidative stress due to asbestos is the main cause of increased 8-isoprostane in EBC.	92 asbestos-exposed subjects were examined (46 women and 46 men).	The control group was represented by 46 subjects (23 men and 23 women), employed as hospital technical workers (gatekeepers, adjuncts and helpers, hospital mailmen, etc.) without occupational exposure.	EBC samples were collected using the EcoScreen. Each subject was asked to breathe through the collection kit for 15 min with more than 2 mL of EBC collected. Samples were immediately frozen after collection (−80 °C)	Measurement of 8-isoprostane in EBC is a promising non-invasive means for assessing the activity of asbestos-induced diseases.	20
Sood, 2013, USA, Ref [89]*	To evaluate EBC 8-isoprostane concentrations following allergen-induced bronchoprovocation in asthma.	Eight mild atopic asthmatics (5 women)	Six healthy controls(four women): the majority of enrolled subjects were premenopausal overweight women(age mean ± SD 39.9 ± 9.7)§	EBC was collected using an R-tube and condensate was collected during a period of 20–30 min. EBC was stored at −70 °C.	EBC 8-isoprostane concentrations do not acutely change following bronchoprovocation in subjects with mild asthma.	20
Vizcaya, 2013, Spain, Ref [95]	To evaluate associations of domestic and occupational use of cleaning products with asthma and biomarkers of respiratory health.	42 cleaners with a history of asthma and/or recent respiratory symptoms (participation rate 60%).	53 symptom-free controls (participation rate 44%)	EBC was collected using an EcoScreen condenser. Collection was performed from 09:00 to 10:00 in the morning. Each subject was asked to breathe into the device for 10 min while wearing a nose clip. The samples were stored at –70 °C.	Asthma in cleaning workers is characterized by non-reversible lung function decrement and increased total IgE.	21
Zhao, 2008, Japan, Ref [98]*	the relationship between the pH of EBC and the concentration in EBC of a marker of oxidative stress, 8-isoprostane, was investigated. The relationship between these markers and lung function was also studied.	Adults aged 18 years or over with asthma were recruited (*n* = 44, 20 females, nonsmokers)§	Sex-matched and age-matched healthy volunteers without respiratory disease were recruited as control subjects (*n* = 20, 8 females, nonsmokers).	EBC was collected using an EcoScreen condenser. Collection was performed from 09:00 to 10:00 in the morning. Each subject was asked to breathe into the device for 10 min while wearing a nose clip. The samples were stored at –70 °C.	Stress and oxidative stress assessed by pH and 8-isoprostane concentration, respectively, in EBC did not show parallel changes associated with asthma and were not correlated with lung function in asthma patients.	20

* Studies included in the meta-analysis; § Subgroups included in the meta-analysis; Note. IL-6 = interleukin-6, TNF-a = Tumor Necrosis Factor-alpha, LTB4 = Leukotriene B4, DPT = diffuse pleural thickening, PPs = pleural plaques, OSA = Obstructive sleep apnea, ROS = reactive oxygen species, RNS = Several reactive nitrogen species, PF = pulmonary fibrosis, SDB = Sleep-disordered breathing, nGER = nocturnal gastroesophageal reflux, OSAS = sleep apnea–hypopnea syndrome, GMAW = gas metal arc welding with solid wire, FCAW = flux cored wire, HRCT = high-resolution computed tomography, and IgE = immunoglobulin E.

**Table 2:** 8-isoprostane reference ranges [pg/mL] in the exhaled breath condensate (EBC) of healthy adults*.

EBC Device	Males Only	Males and Females	All Population
**Rtube**	6.23(1.75–10.71), (*n* = 2)	6.36(2.46–10.74), (*n* = 6)	9.26(2.46–10.71), (*n* = 9)
**Ecoscreen**	18.7(8.6–23.0), (*n* = 17)	8.00(4.13–14.23), (*n* = 35)	9.44(5.73–19.15), (*n* = 52)
**NA/Other**	2.81(2.81–2.81), (*n* = 1)	15.41(6.12–19.52), (*n* = 11)	15.37(5.63–19.20), (*n* = 12)

* Results are presented as median (Interquartile range (IQR)), number of subgroups (*n*), only one subgroup had females only and the median was 9.26 pg/mL but no variability can be estimated.

## Supplementary Materials

Supplementary materials can be found at https://www.mdpi.com/1422-0067/21/11/3822/s1.

## Abbreviations

CV	Coefficient of variation
OS	Oxidative stress
EBC	Exhaled Breath Condensate
GM	Geometric Mean
GSD	Geometric standard deviation
BMI	Body Mass Index

## Appendix A

**Table A1:** Mixed effect regression analysis to investigate the relation between levels of 8-isoprostane in EBC [pg/mL] and device used during collection, gender, BMI, smoking status, and mean age of the population.

LogGM	Regression Coefficient	*P* > |z|	95%CI
Device			
Ecoscreen	0		
NA/others	0.64	0.03	0.06–1.21
Rtube	−0.70	0.13	−1.60–0.21
BMI			
Gender			
Males only	0		
Males and females	−0.39	0.38	
BMI<25	0		
BMI>25	−0.19	0.71	−1.25–0.85
No BMI reported	−0.08	0.87	−0.88–1.04
Smoking			
Non-smokers	0		
Smokers and nonsmokers	−0.24	0.43	−0.86–0.37
Smokers	0.10	0.76	−0.56–0.76
Mean age			
<40	0		
40–60	0.02	0.94	−0.48–0.52
>60	0.02	0.96	−0.82–0.87
Intercept	2.31	0.00	1.26–3.36
Between-study standard deviation	0.56		0.25–1.17
Within-study, between-group standard deviation”	0.55		0.29–1.02

Seven studies comprising nine subgroups are excluded from this analysis because of missing values. * *p* < 0.05 corresponds to chi-square test.

**Table A2:** Mixed effect regression analysis to investigate the relation between levels of 8-isoprostane in EBC [pg/mL] and device used during collection, BMI, smoking status, and mean age of the population.

LOGGM	Regression Coefficient	*P* > |z|	95%CI
Device			
Ecoscreen	0		
NA/Other	0.47	0.113	0.11–1.05
Rtube	0.69	0.098	1.5–0.12
Temperature			
−20	−1.31	0.219	−3.40–0.78
−60	−0.12	0.984	−1.26–1.24
−70	0		
−80	−0.14	0.565	−0.66–0.36
NA	−2.73	0.007	−4.73–(−0.73)
Nose-clip			
No	0		
Yes	−0.55	0.119	−1.250.14
Duration			
10	0		
10–15	0.35	0.539	−0.77–1.48
15	−0.07	0.767	−0.55–0.40
20	0.36	0.550	−0.81–1.52
Time			
Afternoon	−0.47	0.431	−1.64–0.70
NA	−0.44	0.12	0.1–0.12
Morning	0		
