# Supplementary material for: Reference Ranges of 8-Isoprostane Concentrations in Exhaled Breath Condensate (EBC): A Systematic Review and Meta-Analysis
Source: Int J Mol Sci. 2020 May 28;21(11):3822. doi: 10.3390/ijms21113822 (PMC7311981; doi:10.3390/ijms21113822)
Supplement: Supplementary file 1 [file ijms-21-03822-s001.pdf]

International Journal of *Molecular Sciences*

MDPI

Table S1. Description of the included studies and the quality assessment results.

Author, year, country, [Ref]*	Study objectives	Population studied and number of participants	Control population and sub-population	Method of EBC collection and analysis	Main findings	Quality score and level
Alfaro, 2007, USA, Ref 15	To determine if a relationship exists between O3 induced pulmonary function changes and the presence of inflammatory markers as measured in EBC samples obtained from O3-sensitive and nonsensitive human subjects.	O3-sensitive healthy adult volunteers(n=4, 2 males and 2 females).	O3-nonsensitive healthy adult volunteers(n=4, 2 males and 2 females).	EBC was obtained via a Jaeger EcoScreen system while wearing a noseclip. Each sampling period lasted 20 minutes and the frozen EBC samples were immediately stored at -80°C.	The results indicate that sensitive subjects have elevated arachidonic acid metabolites in EBCs compared to nonsensitive subjects after O3 inhalation.	11 Low

Andrianjafimasy, 2017, France, Ref 17*	To investigate the associations between biomarkers of response and damage related to oxidative stress, measured from different biological compartments, and asthma outcomes.	EGEA is a French cohort study with three surveys over 20 years. The first EGEA survey (EGEA1) included cases with asthma, recruited in five chest clinics, their first-degree relatives and population-based controls, recruited in the early 1990s in five French cities (n=2047). A follow-up of the participants was completed in 2003–2007 (EGEA2), including 1601 subjects. The present analyses used data collected at EGEA2 (n=1388).	774 participants without ever asthma, 54.4% were females and their mean age ±SD (46.2±15.9). 20.7 % were current smokers.§	EBC was collected using an RTube. After 15 min, EBC was immediately separated in aliquots and stored at −80°C according to standardised procedures.	EBC 8-isoprostanes seem to be involved in childhood-onset asthma and FlOPs (fluorescent oxidation products) seem to be linked to asthma expression and control in adults.	19 Moderate
Antczak, 2002, Poland, Ref 19*	To determine whether cys-LTs, PGE 2, LTB4 , and 8-isoprostane levels are increased in EBC in patients with AIA (aspirin-induced asthma) compared with patients with	31 patients with AIA (mean age, 41±23 years; 23 female, nonsmokers), 26 aspirin-tolerant patients with mild to moderate asthma (ATA) (mean age, 47±18years; 15 female, nonsmokers).	16 healthy subjects (mean age, 45±17 years, 7 female) had no history of any respiratory symptoms or respiratory infection.§	EBC was collected in a tube installed in a polystyrene foam container filled with dry ice. Patients were asked to breathe out spontaneously for 10–15 minutes. Subjects wore a	Cys-LTs and 8-isoprostanes are elevated in EBC of steroid-naive patients with AIA, and that cys-LTs are decreased	12 Low

	ATA (Aspirin-Tolerant Asthma) and healthy subjects and whether there are any differences between steroid-naive and steroid-treated patients with AIA.			noseclip and samples were stored at 80°C.	in steroid-treated patients.	
Antczak, 2011, Poland, Ref 21*	To compare the contents of lipid mediators in EBC and BAL fluid in various patients undergoing bronchoscopy.	37 patients undergoing bronchoscopy for clinical reasons (12 sarcoidosis, 12 COPD, 5 chronic cough, 6 lung cancer, ca planoepitheliale, 1 Wegener's granuloma and 1 sclerodermia) were included.	Ten healthy age-matched volunteers were also included as a control group.§	EBC was collected using Ecoscreen (Jaeger, Germany). Patients were asked to breathe out spontaneously for 15 min. Each subjectwore a noseclip. Samples were stored at −80◦ C.	To compare EBC and BALF in different lung diseases which demonstrated significant correlations between the levels of eicosanoids in BALF and EBCin patientswith COPD and sarcoidosis. EBC may be useful inmeasuring inflammation in several inflammatory lung diseases.	13 Low

Antczak, 2012, Poland, Ref 18*	To determine eicosanoids, 8-isoprostane and H2O2 at various time points in COPD patients hospitalized for an infectious exacerbation of COPD and treated with antibiotics, and to investigate the relationship between these inflammatory markers and clinical variables.	16 patients with severe infectious exacerbations of COPD (mean age 64 ±12 years, 13 male).	13 healthy age-matched controls (mean age 57 ±19 years, 10 male) were recruited for this study.§	EBC samples were obtained using a condensing tubing system, patients were asked to breathe through the collection tube for 15 min. The samples were stored at –80°C.	Eicosanoids and oxidants are increased in infectious exacerbations of COPD. They are also elevated in the airways of stable COPD patients compared to healthy subjects.	12 Low
Ashmawi, 2018, Egypt, Ref 22*	To measure 8-isoprostane levels in EBC of COPD patients and to evaluate the relation between EBC 8-isoprostane and the clinical and functional parameters of COPD.	80 COPD patients.	20 healthy controls (age 41.8±7.1, 16 males, 45% smokers).§	A specially designed condenser was used to collect EBC samples, participants had been informed to breathe tidally for 15 min without a noseclip. EBC was obtained and stored at −70°C.	8-Isoprostane is considered a sensitive and valuable biomarker measured in EBC, reflecting the intensity of the inflammatory process in COPD. 8-Isoprostane showed a significant	18 Moderate

					positive correlation with COPD GOLD stage and negative correlation with lung function.	
Barreto, 2006, Italy, Ref 23	To measure exhaled biomarkers and test lung function in ship engineworkers before and after an 8-hour patrol at sea, during which they lived in restricted quarters in close contact with powerful high-speed diesel engines.	18 healthy volunteer engine-workers serving in the Italian Army (men, mean age 39.7±7.6 years, range 23-56 years) who had no history of respiratory diseases, normal spirometric values and engine-work engagement lasting a similar time. 8 participants were current smokers (smoking history, 19.4 packyears).	Non-smoking healthy volunteer engine-workers serving in the Italian Army. They were 10 non-smokers (men, mean age 41.8, range 23-58 years).	EBC was collected using a specially designed condenser (EcoScreen®, Jaeger, Wiirzburg, Germany). Subjects were asked to breath tidally for 15 minutes. EBC was collected in vials that were stored immediately at -80°C.	The findings suggest that smoking is a risk factor for airway inflammation in ship engine-room workers.	14 Moderate

Biernacki, 2003, UK, Ref 25	To assess markers of inflammation (LTB4) and oxidative stress (8-isoprostane) in EBC in patients with exacerbations of COPD and after treatment with antibiotics.	30 patients (19 men) with exacerbations of COPD were recruited from general practice clinics.	12 control subjects (8 men) were recruited from the staff working in the practices. All were non-smokers and had no significant past medical history.	EBC was collected by an Eco Screen condenser (Jaeger, Hoechberg, Germany). Patients were required to breathe tidally for 10 minutes while wearing a nose clip. Samples were stored in the laboratory at –70°C.	Non-invasive markers of inflammation and oxidative stress are increased during an infective exacerbation of COPD and only slowly recover after treatment with antibiotics.	14 Moderate
Borrill, 2008, UK, Ref 26	To compare pulmonary function and non-invasive biomarker data between smokers and non-smokers to gain a comprehensive understanding of the early physiological and inflammatory effects of cigarette smoking, and to investigate the relationships between non-invasive	18 current smokers (mean age 46.4 [SD 9.6], 7 male, mean pack years 25.5 [SD 10.3])	Ten lifelong non-smoking healthy controls (mean age 44.8 [SD 15.6], 4 male).	EBC was collected during tidal breathing for 10 minutes (EcoScreen, Jaeger, Hoechberg, Germany) without a nose pe. Samples were frozen at −80 ºC.	Smokers had evidence of small airway dysfunction, increased airway resistance, reduced lung compliance, airway neutrophilia and oxidative stress.	11 Low

	biomarkers in smokers.					
Brindicci, 2009, UK, Ref 27*	To investigate the effects of aminoguanidine on JNO and concentration of NO in peripheral lung (CALV) in COPO patients compared to healthy smokers and nonsmokers. The effect of aminoguanidine on NO products in EBC and in sputum was also investigated together with the effects on 8-isoprostane.	Ten patients with COPD.	Ten healthy smokers had no history of respiratory disease, had normal spirometry results, and had a smoking history> 10 pack-yrs. 10 healthy nonatopic nonsmokers with normal lung function were also enrolled.§	Subjects were asked to breath tidallv through a mouthpiece connected to the condenser (EcoScreen) while wearing a nose clip for a period of 10 min. EBC samples were stored at -80°C.	The results suggest that the constitutive NOS isoform as well as iNOS might be involved in NO release and contribute to the high CALV and ONOO- production in patients with COPD.	15 Moderate

Brussino, 2010, Italy, Ref 28*	To investigate oxidative stress induced by allergen challenge in mild asthmatics, by measuring 8-isoprostane in EBC, and to examine itsrelationship with lipid mediators derived from arachidonic acid, cys-LTs and PGE2.	Patients: 12 non-smoking patients, sensitized to cat, with mild intermittent asthma.	20 normal subjects matched for age (mean age 32, range 20–56 years) and gender (six male) acted as controls for baseline values.§	EBC collection was performed using the R Tube™. EBC collections were obtained after breathing for 10min and samples were immediately stored at 80°C.	The increase in EBC 8-isoprostane observed after allergen challenge indicates that allergen exposure increases airway oxidative stress in allergic asthma. The strict correlation between cys-LTs and 8-isoprostane underlines the relationship between allergic inflammation and oxidative stress.	15 Moderate
Carpagnano, 2002, Italy. Ref 29	To analyze the presence of 8-isoprostane, and interleukin (IL)-6, in EBC of OSA patients and subjects matched for obesity in order to see whether these	The study population consisted of 18 OSA patients and 10 subjects matched for obesity who did not have OSA.	The group of control subjects consisted of 15 subjects (eight men; mean [SEM] age, 42[4]years), of normal weight (body mass index, < 27 kg/m^2), with no sleep	EBC was collected at 8 am by using acondenser (EcoScreen). Subjects were asked to breathe at a normal frequency and tidal volume, wearing a nose clip, for a period of 10 min.	Inflammation and oxidative stress are characteristic in the airways of OSA patients but not in obese subjects, and that their levels	17 Moderate

	markers reflect the severity of OSA and whether they could be used to screen obese subjects with a high risk of developing OSA.		disturbances, and with good health.§	EBC samples was stored at 80°C immediately.	depend on the severity of the OSA.	
Carpagnano, 2004, Italy, Ref 30*	To investigate whether short term supplementary oxygen (28%) increases oxidative stress and inflammation in the airways we measured 8-isoprostane and IL-6 concentrations in EBC of healthy subjects and COPD patients after breathing room air and then after exposure to 28% oxygen.	23 COPD patients who were all confirmed ex-smokers were studied.	23 healthy subjects were non-smokers who had no respiratory symptoms or respiratory tract infection for >3 months before the study. Healthy subjects who were receiving oxygen were 18 and 5 were receiving air.§	EBC was collected using a condenser (EcoScreen), with tidal breathing for 10 minutes. Condensate was collected on ice and stored at -70˚C.	The findings suggest that short term supplementary oxygen may enhance oxidative stress and inflammation in the airways. Whether this happens with long term oxygen therapy needs to be determined.	15 Moderate

Chan, 2009, Australia, Ref 31	to measure specific oxidative stress markers inthe EBC of subjects, including H2O2, 8-isoprostane, pH, and antioxidant capacity.	Patients with newly diagnosed lung cancer before treatment were recruited from the oncology clinic (n=21).	The control group comprised nonsmokers (n=21), smokers (n=16), and exsmokers(n=13), defined as not having smoked for at least 1 year. The control group included subjects without lung cancer, and no history of chronic obstructive pulmonary disease (COPD) or other respiratory conditions matched for socioeconomic and age group.	EBC was obtained by breathing into a nonsiliconized glass collection device. Subjects breathed tidally for 10 to 15 minutes The samples were stored under argon at -80°C.	The findings suggest oxidative stress is implicated in the development of lung cancer and may be an early marker of the disease.	15 Moderate
Ciebiada, 2012, Poland, Ref 34*	To assess the levels of cys-LTs, PGE2, LTB4 and 8-isoprostane in the EBC of patients with primary lung cancer and to compare with those detected in the bronchoalveolar lavage fluid (BALf) of patients diagnosed with NSCLC and those	17 included patients with diagnosis of lung cancer.	Ten healthy smokers controls(5 male, mean age±SD 49.9 ± 15.2) and 12 healthy nonsmokers controls(7 male, mean age±SD 37.8 ± 13.1).§	Patients were instructed to breathe for 10–15 minutes while wearing a nose clip. The condensate was collected by a commercially available condenser (EcoScreen). Condensate was immediately stored at -80◦ C.	Since cys-LT, LTB4 and 8-isoprostane concentrations in EBC from patients with lung cancer reflect their concentrations in BALf, they may serve as a possible non-invasive	15 Moderate

	detected in EBC of healthy control.					method to monitor the disease and to assess the effectiveness of therapy.	
Cruz, 2009, Spain, Ref 35	To determine whether there are significant ageassociated differences in pH values, 8-isoprostane, and nitrogen oxides in EBC from a population of healthy adults in different age groups.	NA		75 healthy, nonsmoking subjects participated in the study. Subjects werestratified into five age groups: 18 to 29 years (group 1),30 to 39 years (group 2), 40 to 49 years (group 3), .50 to .59 years (group 4), and 60 to 80 years (group .5). Each group has 15 subjects.	EBC was collected using EcoScreen). Subjects breathed tidally while wearing nose clips. The aliquots, used for measuring nitrite, nitrate, and 8-isoprostane were immediatelywere stored at -70°C.	The results indicate that pH and 8-isoprostane levels in EBC show a relationship with age. Thus, values obtained in studies with control groups may require adjustment for these factors.	16 Moderate

Do, 2008, Canada, Ref 36	To evaluate the relationship, among grain elevator workers, between EBC biomarkers of airway acidity (pH and NH4) and oxidative stress (8-isoprostane) on the one hand, and personal characteristics and work exposures on the other.	This study was nested in a larger cross-sectional survey of employees in five terminal grain elevators. There were 82 eligible participants; 4 did not show up for testing and 3 were excluded from analysis because of missing exposure measurements, leaving 75 participants for this analysis (6 females, 15% smokers, mean age=47).	NA	EBC collection was performed using the R-Tube. EBC collection was performed for 15 minutes at a tidal breathing rate.	Chronic exposures are associated with airway acidity, whereas acute exposures are more closely associated with oxidative stress. The collection of EBC may contribute to predicting the pathological state of the airways of workers exposed to acute and chronic factors.	18 Moderate
Alvarez, 2016, Spain, Ref 16*	To measure the concentration of 8-isoprostane as a biomarker of OS in a group of patients with obstructive sleep apnea–hypopnea syndrome (OSAS), and	The cases (n=30) were consecutive patients with OSAS diagnosed.	The control group (n=12, 7 females, mean age±SD 42±7) comprised non-smoking patients without prior respiratory, local or systemic inflammatory disease.§	EBC was collected using the EcoScreen II device. The technique is performed in a sitting position, with a nose clip and tidal breathing for 15 min. The samples were stored at -70°C.	Snoring, and not OSAS severity, could be the phenomenon underlying the presence of local OS measured in the airway of	15 Moderate

	compared with a control group.					patients with OSAS.	
Font-Ribera, 2010, Spain, Ref 38	To explore short-term respiratory changes in healthy adults after swimming in an indoor chlorinated swimming pool by measuring lung function and a wide range of biomarkers that may reflect different mechanisms of effect.	50 subjects were recruited for the study, 2 subjects were excluded with history of asthma for the present analysis, resulting in a sample of 48 subjects. Most participants were women (65%) and were highly educated (92% with university studies), with an average age (± SD) of 30 ± 6.1 years.	NA		EBC was obtained using an EcoScreen condenser Samples were obtained through breathing at normal frequency and tidal volume until a total expiratory volume of 180 L was achieved. All samples were lyophilized and stored at –80°	We detected a slight increase in serum CC16, a marker of lung epithelium permeability, in healthy adults after they swam in an indoor chlorinated pool. Exercise and DBP exposure explained this association, without involving inflammatory mechanisms.	15 Moderate

Goldoni, 2013, Italy, Ref 39*	To compare the concentration of several biomarkers in traditionally collected whole EBC (W-EBC) and in fractionated EBC (A-EBC), in which only the exhaled air with a CO2 concentration above C50 of the saturation value is condensed. A specific device was designed to perform it.	NA	Enrolled subjects were 45 (6 smokers, 39 nonsmokers), they did not present any pulmonary symptom or a history of pulmonary disease, and all had normal lung spirometry.§	EBC collection was performed with TURBO-DECCS, the subjects were asked to tidally breath inside the condenser for 15 min without a nose clip. Samples were aliquoted and stored at -80 ◦C.	The fractionation of exhaled air may be promising in clinical and occupational medicine.	14 Moderate
Gratziou, 2008, Greece, Ref 40*	To investigate breath markers of as and airway inflammation in patients with seasonal allergic rhinitis (SAR) with and without concomitant asthma.	23 patients with a history of SAR for the last two years took part in the study.	Healthy subjects (3 males, mean age ±SD 36 ± 8, nonsmokers)§	EBC was collected using EcoScreen, subjects were asked to breathe at normal frequency and tidal volume, wearing a nose clip, for a period of 15 min and aliquots were stored immediately at -80°C.	OS markers were decreased at normal levels out of pollen season. Natural allergen exposure induces OS and airway inflammation in patients with SAR who have no clinical signs of lower airway involvement.	14 Moderate

Hakim, 2011, Isreal, Ref 41	To evaluate the short-term effects of a single, 30-min session of water pipe smoking (WPS) on COHb levels and cardiorespiratory and airway inflammatory parameters in volunteers.	Eligible subjects were older than 18 years and had previously smoked from WPs. 45 subjects (30 men, 15 women) were included. Their mean age±SD 32.35±23.36, Cigarettes smokers (n=8).	NA	EBC samples were collected in Rtubes. The collected EBCs were stored at -80°C until analysis.	one session of WPS causes acute biologic changes that might result in marked health problems. It adds to the limited evidence that WPS is harmful and supports interventions to control the continuing global spread of WPS, especially among youth.	15 Moderate
Heinicke, 2009, Chile, Ref 42*	To determine oxidative stress response parameters upon exercise or rest during a 6-week-sojourn at moderate altitude (2,800 m).	Ten Athletes (four females and six males; mean age±SD, 24.7 ± 1.3 years), who usually live at sea level.§	Five sedentary control subjects (five males, 30.2 ± 3.3 years), who usually live at sea level.§	EBC was collected with a self-constructed device.EBC was collected during a period of 20 min and immediately stored in liquid nitrogen until analyzed.	A stay at moderate altitude for up to 6 weeks increases markers of oxidative stress in EBC independent of additional endurance training. Notably, this oxidative	10 Low

					stress is still detecTable 3 days upon return to sea level.	
Hoffmeyer, 2009, Germany, Ref 43*	To compare the temperature-controlled ECoScreen2 with ECoScreen.	NA	16 non-smoking healthy volunteers, 9 males, age median(range) 39(26-62)§	EBC was collected in each subject between 8 and 12 a.m. In this study the compared We compared ECoScreen and ECoScreen2. The subjects used a nose-clip and EBC was collected for exactly 10 min. Samples were stored at -70°C.	ECoScreen2 in combination with mediator specific enzyme immunoassays may be suitable for measurement of different biomarkers.	11 Low
Marcin Kazmierczak, 2015, Poland, Ref 46*	To compare the EBC and serum concentrations of CRP, IL-8, LTB4 and 8-isoprostane in COPD patients with those of healthy individuals, and to assess their concentration sin COPD patients with	24 COPD patients with CVD were allocated to group A(3 females, mean age ±SD 67.75±9.3) and 20 COPD patients without CVD were assigned to group B (10 females, mean±SD 65.05±8.0).	16 healthy individuals as controls (12 femles, mean age±SD 53.1±7.31)§§	EBC was collected using the EcoScreen. Patients breathed for 10-15 minutes. Condensate was immediately frozen and stored at -20°C.	Although systemic inflammation coexists with COPD,it is not elevated in COPD patients with CVD.Since this phenomenon may result from	14 Moderate

	CVD and those without cardiovascular complications.				treatment with statins, future studies should state whether COPD patients could benefit from the additional statin therapy.	
Ko, 2006, Hong Kong, Ref 47*	To assess the level of oxidative stress and inflammation in the airway non-invasively by collection of EBC. The levels of 8-isoprostane, GROa and MCP-1 were measured in both COPD patients and controls and the relationship between their levels, lung function and dyspnoea score was assessed.	32 COPD patients (28 males, mean±age 72±9, exsomkers) were recruited.	Age and sex matched non-smoking subjects with no known chronic respiratory diseases.(13 males, 73±5, nonsmokers)§	EBC was collected using EcoScreen, the collection was done from 9 to 10 am in the morning. Each subject was asked to breathe through the collection kit for 10 min while wearing a noseclip. EBC was stored immediately at -70°C.	8-isoprostane level, but not GROa and MCP-1, in EBC was increased in COPD patients with poorer lung function. This suggests an increased oxidative stress in the airway in patients with more severe COPD.	19 Moderate

Koskela, 2012, Finland, Ref 49*	To investigate the associations of these levels with cough related quality of life and cough sensitivity to hypertonicity and hyperpnoea .	32 subjects with stable asthma were recruitedwere recruited. All subjects completed the study but due to technical reasons, EBC analysis could not be performed in six asthmatic subjects. Thus, the present analysis consists of the results of 26 asthmatic (7 males, mean age±SD 42±3, 7 smokers) .	Ten Healthy subjects (2 males, mean±SD 39±6, nonsmokers)§	EBC was collected utilizing Ecoscreen. The subjects wore a nose clip. The duration of collectio was 10 min, using tidal breathing. The condensate was immediately stored in −70◦C.	Airway oxidative stress may be associated with experienced cough severity and measured cough sensitivity in asthma.	16 Moderate
Koskela, 2013, Finland, Ref 48*	To investigate whether airway oxidative stress is present also in subjects without doctor's diagnosis of any chronic lung disorders but who seek medical advice due to chronic cough.	43 subjects with chronic cough (32 females, mean age 55.6).	15 healthy, non-smoking subjects (11 females, mean age 52.7)§	EBC was collected utilizing Ecoscreen. The subjects sat and wore a noseclip. The duration of collection was 10 minutes, the condensate was stored in – 70°C.	Chronic cough seems to be associated with airway oxidative stress in subjects with chronic cough but without chronic lung diseases. This finding may help to develop novel antitussive drugs.	15 Moderate

Koskela, 2015, Finalnd, Ref 50*	To assess whether dust exposure in foundry work is associated with airway inflammation. Inflammation was assessed by measuring alveolar and bronchial NO output and analyzing levels of inflammatory markers in EBC (8-isoprostane and LTB4) and in serum samples (MPO, E-selectin, IL-6, IL-8, IgE, and CRP).	A cross-sectional study was conducted in two Finnish foundries of the same company: one was a steel cast foundry and the other an iron cast foundry. The study population consisted of 476 people: 322 exposed (males). The participation rate was 78%.	Control Persons (154 unexposed workers): Age Mean (SD) 44.1 (9.7), all males. The participation rate was 78%.§	EBC was collected during 15 min of tidal breathing by the Ecoscreen condenser while wearing nose clips. The samples were stored at −70◦C until assayed.	Dust exposure in foundry work may induce both systemic and alveolar inflammation.	17 Moderate
Kostikas, 2002, Greece, Ref 51*	To determine the amounts of pH in the expired breath condensate of patients with asthma, COPD, and bronchiectasis. They investigated the relationship between pH and the inflammatory process, oxidative stress,	Patients with asthma(n=40), COPD (n=20), and bronchiectasis (n=20).	Control Subjects(n=10): All normal subjects were nonsmokers and had a negative history of allergy, their age mean(SD): 34 (8), 7 males and 3 females.§	subjects were comfortably seated in a chair. They were wearing nose clips and breathed in a relaxed manner (tidal breathing) for 15 minutes. Samples were and stored in -70°C.	The pH of the expired breath condensate might be a simple, noninvasive, inexpensive, and easily repeatable procedure for the evaluation of the inflammatory	15 Moderate

	metabolism, and lung function tests.				process in airway diseases.	
Kurti, 2017, USA, Ref 52*	To assess the postprandial airway and systemic 8-isoprostane responses to a high-fat meal (HFM) and a more true-to-life moderate-fat meal (MFM) in insufficiently active male participants.	NA	Eight college-aged males who did not engage regularly in any planned physical activity per week, and did not meet physical activity guidelines (moderate-to-vigorous physical activity (MVPA) less than 150 minutes per week.§	RTubes were used for EBC measurements. Participants performed tidal breathing for 10 minutes while seated, with feet flat on the floor and wearing a nose-clip. Samples immediately frozen at -60°C.	Following a HFM and MFM, 8-isoprostane increases systemically, however airway 8-isoprostane does not change.	14 Moderate
Kurti, 2018, USA, Ref 12*	To determine whether changes in airway 8-isoprostane generation were correlated with changes in lung function. We hypothesized that older women (OW) would have elevated airway 8-isoprostane responses from pre- to	§36 individuals were recruited by age to participate in the study (12 OW, 12 OM, and 12 YC). The OW were post-menopausal and matched for age with the OM and for habitual physical activity level with the OM and YC.§	Young controls (6 males, mean age±SD 21.5 ± 2.3), Older women (n=12, mean age±SD 64.1 ± 4.1) and older men (n=12, mean age±SD 66.2 ± 4.1).§	Subjects performed tidal breathing for 10 minutes into an Rtube, the samples were frozen in a -60 degree Celsius freezer.	The OW exhibited a greater airway 8-isoprostane response to exhaustive exercise compared to OM and YC, which may suggest that sex differences in oxidative stress	13 Low

	post-exercise compared to older men (OM) and younger control (YC).				generation following exhaustive exercise may provide a mechanistic rationale for sex differences in late-onset respiratory diseases.	
Lehtonen, 2007, Finland, Ref 54*	To determine whether patients with asbestosis have altered bronchial or alveolar NO output or increased concentrations of inflammatory markers LTB4 and/or 8-isoprostane in their exhaled breath condensate.	15 patients with asbestosis (all men) of mean age 67 years and 15 were recruited. All subjects were non-smokers.	15 age-matched healthy male volunteers of mean age 63 years with normal spirometric values(nonsmokers)§	EBC was collected during 15 min of tidal breathing with Ecoscreen condenser while wearing nose clips. The samples were stored at –70°C.	Patients with asbestosis have an increased alveolar NO concentration and high levels of leukotriene B4 and 8-isoprostane in exhaled breath.	19 Moderate

Leung, 2005, China, Ref 55	To investigate the relationship between EBC pH and clinical, atopic, and spirometric parameters, exhaled nitric oxide levels (FeNO), and EBC concentrations of 8-isoprostane, LTB4, and cysteinyl-leukotrienes (cys-LT) in asthmatic children.	Patients aged 7–18 years, diagnosed with asthma underwent EBC collection using both RTube and EcoScreen.	Nine healthy, nonsmoking adults (five men) aged 23–39 years	EBC was collected using EcoScreen (Jaeger) or Rtubes. Subjects underwent a 5-min EBC collection without wearing a noseclip and samples were stored at -70°.	pH in non-deaeratedEBCis influenced by asthma severity in children. EBCpH measurement is reproducible, but is dependent on the collection devices used.	13 Low
Li, 2007, China, Ref 56*	To identify the best biomarker, either singly or in combination, that can be used to predict severity of OSAS with the best cost-effectiveness ratio.	22 non-OSAS control subjects, 22 mild OSAS, 22 moderate OSAS, and 24 severe OSAS.	22 non-OSAS control subjects (14 males, mean±age SD 43±93) and 10 healthy smoker subject(10 males, mean±age 41±4)§	EBC was collected using a Ecoscreen condenser, after the completion of sleep monitoring in the morning before 8 a.m. Subjects were asked to breathe while wearing a nose clip, for a period of 15 min. Samples were stored at -80°C	Among non-smoker OSAS suspects, EBC IL-6 and IL-10 have potential to predict severity of OSAS.	17 Moderate
Liou, 2017, China, Ref 57	To assess whether global DNA methylation levels, oxidative stress markers of nucleic	87 workers, which comprised of 26 nano-TiO2handling workers, 31 nano-SiO2handling	Non-exposed control workers (n = 43), 18 men and 25 women, their age mean±SD 37.95±8.94.	EBC was collected by ECoScreen and stored at−80◦C until analysis.	Exposure to metal oxide NMs may lead to global methylation, DNAoxidative	15 Moderate

	acids (8-OHdG) in urine and white blood cell (WBC), and EBC 8-isoprostanewere associated with nanomaterial exposure.	workers, 30 ITO handlingworkera.			damage, and lipid peroxidation.	
Makris, 2007, Greece, Ref 58*	To evaluate the concentrations of 8-isoprostane in EBC of patients with stable COPD and to investigate whether there is any relationship between 8-isoprostane levels and main parameters of the disease such as symptoms, stage of severity, emphysematous changes and airway inflammation.	18 patients with COPD (ex-smokers).	12 healthy individuals 3 males and 9 females, their mean age±SD 61±3.5§	EBC was collected using a specially designed double-jacketed glass tube. Subjects, breathed for 10 min, while using noseclips. One ml of breath condensate was stored at – 70 ° C.	EBC 8-isoprostane levels may reflect the extension of lung emphysema in COPD patients. In this respect, further investigation is required in order to evaluate the possible role of EBC 8-isoprostane in assessing disease progress in COPD patients.	18 Moderate

Marie-Desvergne, 2018, France, Ref 59*	To evaluate the suitability and efficiency of the SensAbues (SB) for collecting 8-isoprostane, in comparison with exhaled breath condensate collection using RT.	NA	Seven volunteers were recruited among the laboratory staff. Volunteers were mainly women (six women and one man), non-smokers (5 non-smokers and 2 smokers) and aged 37 ± 12 years. All of them were healthy with no respiratory inflammation or fever reported at the time of the collections.§	The sampling with the RT was performed for 15 minutes, with a nose-clip. The EBC was immediately frozen at -80°C after collection in the RT collection tube.	This is the first study to report the ability of the SB device to collect and measure 8-isoprostane in exhaled breath. The proposed method offers better sensitivity than a classical collection with the RT device and should be further explored before future application in biomonitoring studies.	11 Low
Mazur, 2009, Finland, Ref 60*	To compare the relative proportions of 2 potent biomarkers, IL-8 and 8-isoprostane, in the induced sputum and EBC sampled from the same subjects nonsmokers,	Nine smokers (mean smoking history 52 pack years) with symptoms.§	14 healthy nonsmokers(3 female, mean age±SD 57±4.2) and 17 healthy smokers (5 female, mean age±SD 43±12.6)§	EBC was collected using EcoScreen with patients breathing at tidal volume for 15 min. Condensate was mmediately stored at –80 ° C for subsequent analysis.	The levels of both potential markers were clearly higher in the induced sputum than in EBC. The results point to an advantage of	15 Moderate

	nonsymptomatic smokers and symptomatic smokers who are considered to be at risk for COPD.				induced sputum over EBC for assessing the degree of airway oxidative stress and inflammation in smokers with a potential risk for COPD development.	
Montuschi, 1999, UK, Ref 3*	To investigate whether 8-isoprostane could be detected in breath condensate of asthma patients, and to compare its concentrations in these patients with those in healthy subjects. The use of breath condensate is a noninvasive means for collecting airway secretions	12 patients with mild asthma, 17 with moderate asthma, and 15 with severe asthma were studied	Healthy control subjects (n=10, mean age 34.1, 4 females)§	EBC samples were collected in a specially designed glass condensing chamber. subjects breathed tidally through a mouthpiece connected to the condenser for 15 min while wearing noseclips. Condensate was stored at -70°C.	There was no correlation between 8-isoprostane and lung function tests in any group of patients. Our study shows that oxidative stress is increased in asthmatic subjects as reflected by 8-isoprostane concentrations in breath condensate.	17 Moderate

Paolo Montuschi, 2002, UK, Ref 61*	To investigate if oxidant stress, as reflected by exhaled 8-isoprostane, is affected by short-term ozone exposure and the effect of inhaled budesonide on free radical production after ozone exposure.	NA	Nine healthy subjects were studied (four men and five women; mean age: 30 years)§	9 healthy subjects were studied (4 men and 5 women, all nonsmokers)	Short-term ozone exposure causes acute increase in lung oxidative stress as reflected by exhaled 8-isoprostane. This increase is resistant to pretreatment with a high dose of inhaled budesonide.	14 Moderate
Papaioannou, 2010, Greece, Ref 62*	asthmatic patients. To evaluate the acute effect of cigarette smoking on biomarkers of airway and systemic inflammation and oxidative stress in patients with well-controlled moderate persistent asthma on proper treatment and in properly matched normal smokers.	Ten patients with moderate persistent asthma were included in the study.	Ten otherwise normal smokers, age and sex-matched with similar smoking habit with the asthmatics served as controls. They were normal smokers (4 females, mean age±SD 35.4±14)§	EBC was collected using EcoScreen. Subjects were asked to perform tidal breathing for 20 min while wearing a nose clip. Samples were stored stored in -80°C	Acute smoking has more deleterious effects in well-controlled properly treated asthmatic smokers compared with matched normal smokers.	19 Moderate

Pękala-Wojciechowska, 2018, Poland, Ref 63*	To measure oxidative stress in type 1 diabetes by the concentration of 8-isoprostanes in the exhaled breath condensate.	Patients with type 1 diabetes without complications group (n=10) and type 1 diabetes group with advanced complications (n=11) were included in the study.	The control group consisted of 12 healthy persons (2 men, age median/min-max 34/25-60)§	EBC was obtained for each patient by means of the EcoScreen condenser. Each of the subjects was asked to calmly breathe for 10 minutes using a condenser. The resulting material was frozen at –80 ° C.	Measurement of 8-isoprostanes in the EBC in patients with type 1 diabetes does not appear to be a good diagnostic tool for monitoring the activity of oxidative stress in these patients.	12 Low
Pelclova, 2011, Czech Republic, Ref 67	To evaluate the potential impact of lung fibrosis on the levels of the oxidative stress markers in blood and urine.	37 patients, formerly exposed to silica (36 men, 1 woman, mean age 69.1 ± 2.9; 13.5% smokers).	29 control subjects (20 men, 9 women, mean age 67.0 ± 4.6 yr; 13.8% smokers)	EBC collection was performed with EcoScreen. All subjects (wearing a nose-clip) breathed tidally and samples were immediately frozen to –80°C.	The influence of satisfactorily controlled systemic disorders on markers in EBC in patients with pneumoconioses is not significant. In addition to oxidative stress markers in EBC, lung fibroses may increase oxidative	15 Moderate

					stress markers in plasma and urine.	
Pelclova, 2014, Czech Republic, Ref 68	To search for optimal markers in the EBC, plasma and urine that would reflect the severity of occupational asthma (OA) and distinguish between a stable asthma and asthma with signs of activity.	43 subjects with previously diagnosed immunological OA (18 men, 25 women, mean age: 49.3±11.8 years) after withdrawal from the occupational allergen exposure.	Control group consisted of 20 subjects, working as office or health care employees and having no symptoms of asthma (10 men, 10 women, mean age: 49±14.9 years).	EBC was collected within the following 15–20 min with EcoScreen. All subjects (wearing a nose-clip) breathed tidally and samples were immediately frozen to −80°C.	The improvement in OA is very slow and objective impairments persist years after removal from the exposure. Cysteinyl LTs and 8-ISO in EBC and 8-ISO in plasma might enrich the spectrum of useful objective tests for the follow-up of OA.	18 Moderate

Pelclova, 2012, Czech Republic, 66	To study the possible adverse health-effects in workers exposed to TiO2 aerosol through non-invasive method such as the analysis of markers of oxidative stress and inflammation in EBC and the measurement of FeNO.	20 workers exposed to TiO2 aerosol (males, 11 smokers, 9 nonsmokers).	19 controls (males, 11 smokers, 8 nonsmokers).	EBC samples collected using Ecoscreen. Nose breathing was eliminated by nose clips.	This first in-vivo study in workers suggests adverse effects of chronic exposure to TiO2 aerosol including nano-sized fractions.	17 Moderate
Pelclova, 2016, Czech Republic, Ref 70	To evaluate markers of oxidative stress in the EBC of workers exposed to iron oxide nanoparticles during iron oxide pigment production and their association with workplace environments.	14 workers (males, 43 ± 7 years, 43% smokers) exposure to iron oxides.	The control group was composed of 14 males (39 ± 4 years, 50% smokers), who were not employed in this factory and were not exposed occupationally to dust or other health risks.	EBC samples were collected using Ecoscreen Turbo. All subjects breathed tidally for about 15 min while wearing a noseclip. All samples were immediately frozen and stored at −80 °C.	The adverse effects of nano iron oxide aerosol exposure and support the utility of oxidative stress biomarkers in EBC. The analysis of urine oxidative stress biomarkers does not support the presence of systemic oxidative stress in iron oxide pigment	16 Moderate

					production workers.	
Pelclova, 2017, Czech Republic, Ref 71	To investigate the impact of such short-term exposures on the markers of health effects in office workers relative to production workers from the same factory.	22 male office employees exposed to TiO2 working in the same building.	Seven male control subjects mean age±SD 38.5 ± 4.5 years, they werenot employed in the factory. They worked as healthcare personnel and technical staff and did not handle nanomaterials	EBC samples were collected using Ecoscreen Turbo. All subjects breathed tidally for about 15 min while wearing a noseclip. All samples were immediately frozen and stored at −80 °C.	More attention should be paid to both production and office employees in the factories with nanoparticle exposure, and a more detailed chemical analysis of their workplace aerosol is needed.	16 Moderate

Pelclova, 2017, Czech Republic, Ref 72	To expand the spectrum of investigation to markers of lipid oxidation both in EBC and urine and to identify the most robust oxidative stress markers for routine biomonitoring of exposed workers.	Workers working with TiO_2 for at least 6 months (divided into subgroups).	45 male controls, with a mean age of 34.2 (CI 31.5–36.9) years; 40.0% were smokers and 100.0% were daily alcohol users. They were not occupationally exposed to TiO_2, dusts, or other hazardous substance	EBC samples were collected using Ecoscreen Turbo. All subjects breathed tidally for about 15 min while wearing a noseclip. All samples were immediately frozen and stored at −80 °C.	A significant dose-dependent association was found between exposure to TiO_2 and markers of lipid oxidation in the EBC. These markers were not elevated in the urine samples. Lipid oxidation in the EBC of workers exposed to (nano)TiO_2 complements our earlier findings on DNA and protein damage.	16 Moderate
Pelclova, 2018, Czech Republic, Ref 69	To investigate the information concerning respiratory findings in TCDD intoxication	Eight male survivors (mean age 72.4 ± 1.3 years) from 80 workers intoxicated with TCDD during the production of herbicides from 1965 to 1968.	The healthy control group included 7 men (66.0 ± 16 years), five were smokers (71.4%), and two non-smokers.	EBC samples were collected using Ecoscreen Turbo. All subjects breathed tidally for about 15 min while wearing a noseclip. All samples were immediately frozen and stored at −80 °C.	Differences in the expression of the biomolecular markers in EBC as compared to controls were not associated with lung impairments	13 Low

					and the respiratory parameters measured.	
Piotrowski, 2007, Poland, Ref 73	To assess the differences in levels of eicosanoids (ie, CysLT, LTB4, and 8-isoprostane) in EBC of sarcoidosis patients and healthy subjects.	28 sarcoidosis patients (10 women; mean age, 39.18 years).	17 healthy, nonatopic neversmokers (8 women; mean age, 39.82 years).	EBC was collected using a commercial device (Ecoscreen). Patients were asked to breath out spontaneously for 10 min. All subjects wore a nose clip.<br>Samples were stored at 80°C. The collection of EBC	The positive correlation of EBC 8-isoprostane and BALF CysLT concentrations with the percentage of eosinophils in BALF, and the higher percentage of eosinophils in BALF from patients with grade 3 sarcoidosis, may suggest the possible prognostic value.	18 Moderate

Piotrowski, 2010, Poland, Ref 75	To evaluate the clinical value of EBC 8-Isoprostane in sarcoid patients, followed over a period of 6-12 months.	40 caucasian patients (23 women, age 39 ± 11) with sarcoidosis were include	34 healthy never smokers (19 women, age 45 ± 10), members of a hospital staff, free of respiratory infection in the last 4 weeks.	EBC was collected using a commercial device (Ecoscreen). Patients were asked to breath out spontaneously for 10 min. All subjects wore a nose clip. Samples were stored at 80°C. The collection of EBC	Low initial 8-Isoprostane may be a positive prognostic factor. A decrease of 8-Isoprostane in treated patients reflects a non-specific effect of treatment and is not related to mere regression of disease.	15 Moderate
Piotrowski, 2010, Poland, Ref 74	To evaluate the correlation between both spontaneous and PMA stimulated superoxide anion generation and EBC 8-isoprostane.	29 patients with newly diagnosed, histo-pathologically confirmed sarcoidosis were included.	The control group (20 females, mean age 39.8) consisted of 34 healthy never-smokers, members of the hospital staff and medical students.	EBC was collected using a commercial device (Ecoscreen). Patients were asked to breath out spontaneously for 10 min. All subjects wore a nose clip. Samples were stored at 80°C.	higher concentrations of EBC 8-isoprostane in sarcoidosis and higher spontaneous release of superoxide anion from BALF cells in patients with sarcoidosis. The increase of EBC 8-isoprostane is not	15 Moderate

					directly related to superoxide anion released from BALF cells.	
Piotrowski, 2012, Poland, Ref 76	To evaluate the usefulness of EBC 8-Isoprostane as a marker of severity and control of severe adult asthma.	25 severe, never-smoking asthmatics (age: 51.7 ±9.6 years, 17 women) were studied.There were two control groups: 11 healthy never- smokers constituted a healthy control group and 16 newly diagnosedand never-treated, non-smoking mild asthmatics constituted a never-treated asthma control group (age: 32.0 ±8.5 years, 9 women).	There were two control groups: 11 healthy never-smokers constituted a healthy control group (age: 46.6 ±12.3 years, 5 women)	EBC was collected using a commercial device (Ecoscreen). Patients were asked to breath out spontaneously for 10 min. All subjects wore a nose clip. Samples were stored at 80°C. The collection of EBC	EBC 8-isoprostane measurements are not useful for asthma monitoring.	15 Moderate

Pirozzi, 2015, USA, Ref 78	to determine if naturally occurring episodes of ozone air pollution are associated with increased pulmonary inflammation and oxidative stress, increased respiratory symptoms, and decreased lung function in individuals with COPD compared to controls.	All participants were adults aged 40–85, all nonsmokers. The COPD group(n=11) consisted of former smokers with moderate or severe airflow obstruction.	Control group (n = 9), 5 males, mean age (±SD) 66.8 (±5.6). The control group consisted of former smokers without overt chronic lung disease, airflow obstruction, or emphysema on CT imaging. Non-smoking.	EBC was collected using the R-tube system with tidal breathing for 10 min. Samples were divided frozen at -80 degrees F.	Former smokers both with and without airflow obstruction developed airway oxidative stress and inflammation in association with ozone air pollution episodes.	17 Moderate
Cheryl Pirozzi, 2015, USA, Ref 77*	To determine if former smokers with and without airway obstruction differed in their response to naturally occurring PM air pollution episodes	The COPD group(n=16) consisted of former smokers with moderate or severe COPD, 13 males.	the control group (n=12), 6 males. and a the control group consisted of former smokers without chronic lung disease, airflow obstruction, or emphysema on CT imaging.§	EBC was collected using the R-tube system with tidal breathing for 10 min. Samples were divided frozen at -80 degrees F.	Former smokers with COPD have a distinctive response to particulate air pollution episodes compared to former smokers without airflow obstruction, with increased airway inflammation and	17 Moderate

					respiratory symptoms.	
Psathakis, 2003, Greece, Ref 80*	To measure the levels of 8-isoprostane in the expired breath condensate of patients with sarcoidosis, and to investigate the relation of 8-isoprostane level to disease activity.	30 patients with sarcoidosis and 12 healthy control subjects.	healthy control subjects 5 males and 7 females, mean age±SD 39 ± 9§	EBC was collected in the morning using an alternative way of cooling the tubes. subjects were wearing nose clips and breathed for 10 min. The samples were immediately stored at -70°C	8-isoprostane levels are increased in the expired breath condensate of patients with sarcoidosis and might serve as an index of disease activity.	14 Moderate
Psathakis, 2006, Greece, Ref 79*	To measure the levels of H2O2 and 8-isoprostane, as biomarkers of oxidative stress, in the EBC of patients with idiopathic pulmonary fibrosis (IPF).	16 patients with IPF(9 males, mean age±SD 67 ± 7	healthy control subjects 9 males and 6 females, mean age±SD 56 ± 9§	EBC was collected in the morning using an alternative way of cooling the tubes. subjects were wearing nose clips and breathed for 10 min. The samples were immediately stored at -70°C	H2O2 and 8-isoprostane are increased in the EBC of patients with IPF. H2O2 may be correlated with the severity of the disease in IPF.	14 Moderate

Radulovic, 2015, USA, Ref 81*	To compare levels of biomarkers of inflammation in exhaled breath condensate (EBC) and serum in subjects with chronic tetraplegia, mild asthma, and able-bodied controls.	Patients with tetraplegia (n = 12), asthma (n = 12) were included.	Ten healthy able-bodied controls (all males, mean age±SD 48 ± 10)§	EBC was collected using an EcoScreen EBC collector. Participants were askedto perform resting tidal breathing for 15 min. Samples were immediately stored at −80°.	Through analysis of EBC, levels of 8-IP were significantly elevated compared to levels found in individuals with mild asthma and healthy controls.	16 Moderate
Romero, 2005 , Spain, Ref 82	To determine whether analysis of exhaled breath condensate from patients with severe lung infections reveals changes in the redox state at the airway surface.	48 subjects divided into 4 groups: individuals without respiratory disease (n=14), patients with multilobar pneumonia (n=13), patients who had chronic obstructive pulmonary disease with superinfection (n=14), and mechanically ventilated patients with severe pneumonia (n=7).	The control group (12 males, age±SD 62.86±2.96)was made up of 14 patients admitted for scheduled surgery who had no prior history of respiratory disease, had a normal chest radiograph, and who were either nonsmokers or had given up smoking at least 2 years earlier.	EBC was collected using an ANACON condenser. Collection time was over a period of at least 15 minutes. The aliquots were stored at –80°C prior to analysis.	Analysis of the concentrations of 8-isoprostane and MPO in exhaled breath condensate allows assessment of oxidative stress in the airways of patients with severe lung infections.	12 Low

Rosias, 2006, Netherlands, Ref 84	To investigate the influence of different inner condenser coating surfaces (silicone, borosilicate glass, aluminium, polypropylene, Teflon and EcoScreen1 with a Teflon-like coating) on the measurement of 8-isoprostane and albumin in EBC using both an in vitro and in vivo approach.	NA		28 healthy volunteers age median range 26(20–57), 10 were males and 18 females. 5 were smokers.	Each subject was asked to breathe tidally, while wearing a nose-clip and exhaled for 15 min.Samples were stored at −80°.	A condenser with silicone or glass coating is more efficient for measurement of 8- isoprostane or albumin in exhaled breath condensate, than EcoScreen1, aluminium, polypropylene or Teflon.	14 Moderate
Rosias, 2008, Netherlands, Ref 83*	to assess reproducibility of EBC volume, hydrogen peroxide (H2O2), 8-isoprostane and cytokine measurements using different condensers, including a newly developed glass condenser	NA		30 healthy volunteers age median range 23 (22–36),19 were males and 11 females. 28 were nonsmokers.§	Each subject was asked to breathe tidally, while wearing a nose-clip and exhaled for 15 min.Samples were stored at −80°.	Significantly more EBC volume and biomarker detections were found using the optimised glass condenser. Biomarker reproducibility in EBC in healthy adults was not influenced by the type of condenser.	15 Moderate

Samitas, 2009, Greece, Ref 85*	To investigate baseline values of inflammatory lipid mediators in EBC and their relation to asthma severity	16 mild, 12 moderate and 15 severe asthmatics were studied.	Healthy control subjects(n=19, 12 females, age mean=47)§	EBC was collected in the morning using ECoScreen. All subjects breathed for 10 min not wearing a noseclip. Condensate was immediately stored at 80°C.	8-isoprostane and cys-LT are detectable in EBC of healthy subjects and their levels progressively increase in asthmatic patients according to disease severity.	17 Moderate
Sanak, 2011, Poland, Ref 86	To assess a vast platform of eicosanoids in different asthma phenotypes, including aspirin-intolerant asthma, by means of a recently developed analytic approach based on mass spectrometry.	115 asthmatic subjects , 62 (41 female subjects) of whom had AIA were recruited.	healthy control group(n=38, 12 males, age±SD 43.03±13.1)	Collections of EBC were carried out with the ECO Screen I after tidal breathing for 15 minutes, 2 mL of clear fluid was collected and immediately transferred on ice to a -70°C	The highly sensitive eicosanoid profiling in EBC makes it possible to detect alterations in asthma, especially in its distinct phenotype characterized by hypersensitivity to aspirin and other nonsteroidal anti-	16 Moderate

					inflammatory drugs.	
Santini, 2016, Italy, Ref 87*	Comparing exhaled and non-exhaled non-invasive markers of respiratory inflammation in patients with COPD and healthy subjects and define their relationships with smoking habit.	48 patients with stable COPD who were ex-smokers, 17 patients with stable COPD who were currentsmokers were included	12 healthy current smokers(10 males, mean age 48) and 12 healthy ex-smokers(9 males, mean age 56) were studied.§	EBC was collected using Ecoscreen. Subjects breathed tidally for 15 minutes. EBC samples were stored at -80°C.	the biological meaning of these inflammatory markers depends on type of marker and biological matrix in which is measured.	16 Moderate
Shimizu, 2007, Japan, Ref 88	To determine the usefulness of measurement of the acid stress marker pH and the oxidative stress marker 8-isoprostane by EBC in proton pump inhibitor (PPI) therapy effect on	36 patients with asthma(17 males, mean age 45.2)	26 control subjects(13 males, mean age 46.7)§	EBC was collected using an ECoScreen. The collection was performed from 9: 00 to 11: 00 h in the morning. The subjects were asked to breathe tidally for 15 min, while wearing a nose clip. Samples were stored at –70 ° C.	Measurement of the pH and 8-isoprostane level of EBC may be useful to evaluate the influence of GERD on asthma, as well as to determine the timing of	16 Moderate

	moderate asthma patients with GERD.				intermittent PPI therapy.	
Syslova, 2010, Czech Republic, Ref 91	To test a rapid and easy method for monitoring oxidative stress markers in body fluids of patients with asbestos or silica-induced lung diseases	Patients (n=20) diagnosed with occupational respiratory diseases	The control group (n=10) of healthy non-smokerswas subjects with no medical record of occupational exposure to fibrogenic dusts. Their mean age ±SD 63±5 years and they were all men.	EcoScreen was used to collect the samples of EBC. The subjects wear asked to breathe for 5–10min while wearing a nose-clip. All clinical samples were collected between 8 and 12 a.m. The EBC sample was frozen to -80 ◦C.	The method was tested on samples obtained from patients diagnosed with asbestosis, pleural hyalinosis or silicosis, and then compared to samples from healthy subjects. The difference in concentration levels of biomarkers between the two groups was perceptible in all the body fluids.	13 Low

Syslova, 2009, Czech Republic, Ref 90	A highly selective and sensitive method is presented for the quantification of 8-iso-PGF2α, o-Tyr and 8-OHdG in EBC as significant biomarkers of oxidative stress in vivo. The method was tested on real clinical samples collected from patients diagnosed with asbestosis, silicosis and on the control group of healthy subjects.	Ten patients(male, non-smokers) with occupational lung diseases (either silica- or asbestos-induced disorders due to mean 22 ± 6 years of occupational exposure to silica or asbestos).	Ten subjects(male, non-smokers) without any occupational exposure to fibrogenic dusts.	EcoScreen was used to collect the samples of EBC. The subjects wear asked to breathe for 5–10min while wearing a nose-clip. The EBC sample was frozen to -80 ◦C.	The difference in biomarkers' concentration levels found between the two groups was statistically significant.A significant increase in the 8-OHdG biomarker present in EBC was found in patients previously exposed to carcinogenic minerals asbestos and silica.	15 Moderate
Tanou, 2009, Greece, Ref 92*	To evaluate the impact of smoking on inflammatory and oxidative stress biomarkers in patients with seasonal allergic rhinitis, using non-	40 patients with seasonal allergic rhinitis (20 smokers and 20 non-smokers)	30 healthy subjects (15 smokers and 15 non-smokers)§	EBC samples were collected using an Ecoscreen. Subjects were asked to breathe for 15 min while wearing a nose clip. Samples of EBC were stored at -70°C	Patients with allergic rhinitis present increased LTB4 and 8-isoprostane in their nasal cavity, however, with no significant	19 Moderate

	invasive methods for sample collection.				differences between smokers and non-smokers.	
Tufvesson, 2010, Sweden, Ref 93	To investigate the amount of LTs and 8-isoprostane, a marker of oxidative stress, in EBCfrom SSc patients.	22 SSc patients with median disease duration of 2.1 years were investigated.	16 healthy controls were enrolled	EBC was collected using an ECoScreen. Subjects were asked to breathe tidally for 15 min, wearing a nose clip. The condensates were stored at -70°C.	Increased levels of CysLT and 8-isoprostane in EBC from patients with SSc reflect the inflammatory pattern involving LTs as well as oxidative stress.	14 Moderate
Van Hoydonck, 2004, Belgium, Ref 94*	To determine 8-isoprostane and hydrogen peroxide levels in EBC, and, in addition, to investigate the reproducibility of these measurements.	NA	The study population consisted of 12 male smokers (age range 24–61 yrs; mean±SD 44±14 yrs). All of the smokers were healthy.§	EBC was collected using an ECoScreen. Subjects were asked to breathe tidally for 15 min, wearing a nose clip. The condensates were stored at -80°C.	Levels of 8-isoprostane and hydrogen peroxide cannot be reproducibly assessed in exhaled breath condensate from healthy smokers because of their low concentration and/or the lack of	10 Low

						sensitivity of the available assays.	
Wu, 2019, USA, Ref 96	To determine acute pulmonary responses among the firefighters following the WF smoke exposure.	12 healthy wildland firefighters (9 males and 3 females with an average age of 33 years) were recruited.	NA		EBC was collected using RTube. Each firefighter was instructed to breathe spontaneously for 10 min. The samples were stored at -80°C.	Results show only a marginal cross-shift increase in 8-isoprostane on burn days (.05<p value < .1), suggesting WF smoke exposure causes mild pulmonary responses.	11 Low

Zhang, 2013, China, Ref 97	To examine several prominently hypothesized mechanisms by assessing Beijing residents' biologic responses, at the biomarker level, to drastic changes in air quality brought about by unprecedented air pollution control measures implemented during the 2008 Beijing Olympics.	NA		A total of 137 individuals were screened, from whom 128 nonsmoking healthy subjects (never-smokers) were enrolled in the study. Among these subjects included in the analysis, 119 completed all of the 6 planned visits.	EBC was collected using a Jaeger EcoScreen EBC collector.EBC was collected for 20 minutes, during which time subjects were seated, wearing a nose clip. Samples were immediately stored at −80°C.	Changes in air pollution levels during the Beijing Olympics were associated with acute changes in biomarkers of pulmonary and systemic inflammation, oxidative stress, and hemostasis and in measures of cardiovascular physiology (HR and SBP) in healthy, young adults.	14 Moderate

* Studies included in the meta-analysis; § **Subgroups included in the meta-analysis**; Note. - FlOPs=fluorescent oxidation products, AIA=aspirin-induced asthma, ATA=Aspirin-Tolerant Asthma, LTB4=Leukotriene B4, OSA=Obstructive sleep apnea, PF=pulmonary fibrosis, BALf=bronchoalveolar lavage fluid, nGER=nocturnal gastroesophageal reflux, OSAS=sleep apnea–hypopnea syndrome , BAL=Bronchoalveolar lavage. SAR=seasonal allergic rhinitis, W-EBC=whole EBC , A-EBC=fractionated EBC, WPS=water pipe smoking, DBP=dibutyl phthalate, HFM=high-fat meal, MFM=high-fat meal, CVD=cardiovascular diseases, COPD=Chronic obstructive pulmonary disease, OM=older women, YC=young control, HRCT=high-resolution computed tomography, WBC=white blood cells, RT=Rtube device, SB=SensAbues, OA=occupational asthma, Tio2=Titanium dioxide, IPF=idiopathic pulmonary fibrosis, CT=computed tomography scan, PM=particulate matter, GERD=gastroesophageal reflux disease, PPI=proton pump inhibitor, O-Tyr=oxidized tyrosine, SSc = systematic sclerosis, and WF=wildland fire.

International Journal of
Molecular Sciences
